# To combat poor quality research, eliminate publication requirements

**DOI:** 10.17179/excli2024-7428

**Published:** 2024-06-07

**Authors:** James Stacey Taylor

**Affiliations:** 1 Department of Philosophy, The College of New Jersey, 2000 Pennington Road, Ewing, NJ 08628 USA

## ⁯⁯⁯⁯

In a recent paper in *EXCLI Journal* Bor Luen Tang drew attention to a new form of academic misconduct that is currently underappreciated: The use of AI to generate drafts of scientific manuscripts for publication (Tang, 2023[10]). Tang argued that such use of AI should be considered a form of plagiarism (“AIgiariasm”) and condemned accordingly. Tang's argument is persuasive. It also has further implications for the ethical conduct of research that are just as underappreciated as the practice of AIgiarism: The adverse consequences of relying on quantitative metrics to evaluate research for the purpose of assessing academic productivity in the context of making decisions concerning hiring, tenure, promotion, and the allocation of grants. 

Tang observes that papers generated by AIgiarism could possibly contain erroneous or fictitious references that the LLM (Large Language Model) “hallucinated”. If a paper containing such references were to be published and subsequent researchers who read it did not check it for accuracy it is possible that these erroneous or fictitious references could be repeated in subsequent papers, thus contaminating the scientific literature. 

Tang is correct that AIgiarism could exacerbate the problem of the scientific literature being contaminated by poor quality research and is therefore wrongful. But he does not provide an account of why researchers might resort to AIgiarism. Nor does he offer a solution to this problem. This paper thus serves as a complement to Tang's by rectifying these omissions. 

Why might researchers resort to AIgiarism? The answer seems clear. AI can produce drafts of papers far more quickly than can human researchers. Researchers who desire to increase their publication rate might thus be tempted to use AI to increase the number of academic papers that they could produce in a given time period. They might use AI to produce the first draft and generate a list of references, and then revise the paper to incorporate their experimental results. By having AI perform the initial writing and reference-sourcing such researchers could increase the number of papers that they produce considerably. These papers might include hallucinated references. But these will not necessarily be identified and removed through the process of peer review. Peer reviewers are themselves academics, and so subject to the same pressures to which the researchers who use AIgiarism are subject. Since peer reviews receive little to no professional rewards for their work, they have little incentive to do it well (Taylor, 2022, p. 144[11]). They are thus unlikely to perform the time-consuming task of checking references. They are thus unlikely to identify references that the AI generated in producing the paper that was the result of AIgiarism. Peer review-as it is currently practiced-thus might offer little protection against AIgiarism.

How might the academic community address this problem? Several solutions have been proposed. James Stacey Taylor has suggested offering financial “bounties” to referees who detect empirical errors in the work they are reviewing, with these bounties to be paid to them by the authors whose work contained the errors (Taylor, 2022, p. 186[11]). As Amy E. White has noted, however, this process is likely to disadvantage academics who lack the financial resources to pay these bounties (e.g., academics in the developing world) (White, 2023[13]). The risk of having to pay for such errors will be likely to deter such academics from submitting as much work for publication as they otherwise would have done. This could be a loss to the scientific community as a whole. Jeffrey Carroll has suggested that rather than offering financial incentives to referees journals should instead recognize those referees who have served them well by detecting errors (including erroneous references) in the work they refereed. Carroll suggests that this could be combined with the development of a new professional norm whereby such recognized service is valued similarly to the production of published research (e.g., that critiquing previous findings) by (e.g.,) committees that oversee hiring, tenure, and promotion (Carroll, 2022[2]). Similarly, Leek, Taub, and Pineda suggest that the behavior of referees be made public once the refereeing process is complete (Leek et al., 2011[7]). Pöschl and Koop suggest that the traditional approach of blind refereeing be supplemented by an interactive open-access model of refereeing (Pöschl and Koop, 2008[9]). Submitted articles that pass an initial pre-screening test are thus placed on a journal's website for open discussion. The discussants consist both of invited referees (who post anonymously) and other scientists (who are required to sign their comments). After this process manuscripts are then subjected to standard double-blind peer review. The advantage of this approach is clear: With more scientists critiquing the paper it is more likely that errors (such as those generated by AIgiarism) will be detected. 

Many of these suggested improvements are complementary to each other and could be implemented in conjunction to reduce the flow of flawed research into the pages of journals. But a more radical solution could achieve this end better than all of these approaches combined: Eliminate publication expectations from many academic positions. 

This approach is not as drastic as it appears. Relatively few academic institutions are research intensive institutions; many are primarily concerned with undergraduate teaching. Yet even though their focus is on undergraduate teaching they still require that their faculty publish. They do not, however, require that their faculty publish as much as their colleagues in research institutions, nor do they require that they publish in the same high-profile venues as researchers at research institutions. For example, the Biology Department at a primarily undergraduate university that is ranked first for Top Public Schools in the U.S. News & World rankings for 2024 requires that its faculty publish at least one peer-reviewed scholarly journal article in an “appropriate” journal with another in resubmission to secure tenure and promotion to Associate Professor (Department of Biology, The College of New Jersey, 2021[6]). This relatively low publication requirement is commensurate with the relatively high teaching loads (3/3; six courses a year, three in each semester) to which the faculty at this school are subject. Eliminating the publication requirements at such schools would reduce the flow of flawed research into the pages of academic journals in two ways. 

First, if we assume that the rate of academic misconduct (e.g., the use of AIgiarism) is constant across researchers independently of the type of academic institution they work at, eliminating publication requirements at non-research institutions will reduce the number of flawed articles that are published simply by reducing the overall number of articles that are published. However, it is possible that eliminating publication requirements at non-research R1 institutions will not only reduce the number of flawed articles that are published but will also reduce the percentage of flawed articles that are published. It is plausible to hold that persons who are interested in publishing primarily for self-interested reasons (e.g., to secure tenure, to secure promotion, or to secure a merit raise) would be more willing to cut corners to secure the publications they desire than would persons who are interested in publishing to advance understanding in their field. It is also plausible to hold that the more a person is interested in research rather than teaching the more likely she will be to attempt to secure a primarily research position, while persons who are comparatively less interested in research would be more willing to accept a primarily teaching position. It is thus likely that in aggregate persons who have non-research positions will be comparatively more interested in teaching (and comparatively less interested in research) than persons at more research-orientated positions. If so, then it will be more likely that persons with academic positions at more teaching-orientated positions will be comparatively less interested in research than persons with more research-orientated positions. If there is a correlation between interest in research for its own sake and an unwillingness to cut corners to do it, then give the selection pressures that funnel persons into research or teaching positions, we would expect a higher percentage of self-interested research (i.e., research performed for the purposes of professional advancement) to occur in teaching-orientated institutions rather than their research-orientated counterparts. If such research is correlated with a willingness to cut corners (e.g., through AIgiarism) then eliminating publication requirements at teaching orientated institutions would not only reduce the *amount *of flawed research that is published, but also the *percentage* of flawed research that is published. 

In addition to reducing both the number and rate of flawed research that is submitted for publication eliminating the requirement to publish at such schools would have a further benefit: It would increase the number of students that could be taught by the faculty. Teaching loads at primary teaching institutions with publication requirements are lower than they would be if those requirements did not exist. These lower teaching loads exist in recognition of the time that faculty are supposed to spend on their research. Eliminating the research requirements currently in place would allow those institutions to increase the faculty teaching load, thus enabling more students to benefit from their expertise. 

The suggestion that teaching-orientated institutions eliminate the research requirements they currently expect their faculty to meet raises the question of how faculty who only teach but do no research be evaluated for by their Tenure and Promotion Committees for the purposes of tenure and promotion. An initial response to this question could begin by noting that many institutions of higher education require their teaching faculty to request that students evaluate their teaching. Teaching faculty who applied for tenure or promotion would be required to submit all of the evaluations that they had received during the relevant period of time prior to their application (e.g., since appointment, or since their last promotion). Their tenure or promotion would then be contingent on their having met a certain minimum standard in some or all of the categories in which they were evaluated by their students. 

This initial suggestion for the evaluation for teaching faculty faces several immediate problems. First, student evaluations are often biased against women and persons of color (Mengel et al., 2019[8]; Chávez and Mitchell, 2020[3]). Second, a faculty member's receipt of positive or negative evaluations appears to have less to do with their effectiveness as a teacher than it has to do with extraneous factors, such as their physical attractiveness, nonverbal behavior, or sense of humor (Bryant et al., 1980[1]). 

These concerns about the use of student evaluations in the tenure and promotion of teaching faculty are legitimate. They are especially forceful when used to object to the use of student evaluations to construct *ordinal *rankings of teaching faculty (e.g., to decide who should receive a teaching award). But the suggestion here is not to use student evaluations to *rank *teaching faculty but to determine whether they have met or exceeded a particular *cardinal *threshold. To be sure, these problems with student evaluations will still persist (albeit to a lesser degree) even with this use of them and their biases might at the margins result in persons being denied the tenure or promotion that they had rightly earned. The informational effects of these biases could, however, be mitigated or even eliminated by supplementing these evaluations with further (ordinal) data on the teaching effectiveness of the faculty member in question. If students can decide on their final area of study after they have arrived at the institution of higher education, then they could be polled to determine which faculty members' teaching (if any) motivated them further to study the subject in question or confirmed them in their initial decision. This focus on the subject of the faculty member's teaching would likely elicit responses that would be less affected by the biases that would taint those given by students who might be more focused on the person of the faculty member being evaluated. If a faculty member's positive influence on student interest in her subject measured in this way was comparable to that of her more senior colleagues, then this would support her case for tenure or promotion. Similarly, data on how many students choose to take more than one class with her would also provide objective evidence of her appeal to students in comparison to that of her senior colleagues. 

One could object to these attempts to counter the effects of bias by noting that rather than measuring teaching effectiveness they instead measure how appealing the faculty member's teaching is to students. There are two responses to this objection. The first is that being an appealing teacher is part of being an effective teacher (Collie et al., 2019[4]). The second is that this approach could be supplemented by using further longitudinal data that focuses specifically on the faculty member's teaching effectiveness. Students could be required to complete surveys two years after each of their classes to determine (e.g.) whether they had used anything that they had learned in those classes in subsequent classes or outside the classroom. (Such surveys would not be completed by all students, for some would have graduated or left the institution for other reasons.) Again, such surveys would be intended to focus students' attention away from the person of the instructor and towards the effectiveness of their teaching. 

These suggested approaches to evaluating a faculty member's teaching effectiveness for the purposes of tenure and promotion in the absence any required publication output are, of course, imperfect: They might still evince bias and it is not clear how closely positive evaluations of this sort will correlate with teaching effectiveness. But similar doubts have been raised concerning the correlation of publication with research effectiveness. A junior researcher who has published a sufficient number of papers in sufficiently well-ranked journals to receive tenure might fail to maintain her research productivity. Even if she does continue to publish in well-ranked journals her work might not be of interest outside a very small number of colleagues who work in her niche area and so recommend her work for publication when reviewing it. Would a researcher in such a niche area be as “effective” a researcher as someone who publishes more mainstream work in less well-ranked journals, but who is cited more? We might also ask how citations are to be counted. Although the h-index has become widely used as a proxy for academic productivity its use for this purpose faces serious problems: The databases that it relies upon are inaccurate (Costas and Bordons, 2019[5]), it miscounts the citations of researchers with compound family names (Teixeira da Silva and Dobránszki, 2018[12]), and it does not differentiate between positive and negative citations. Eliminating a research requirement for the tenure and promotion of faculty would thus not necessarily require any more subjective approaches to faculty evaluation than those with which we are already saddled.

Eliminating publication requirements at teaching-orientated schools would have three significant benefits: It would reduce the number of flawed papers that are published, it would reduce the rate of such flawed publications, and it would enable more students to be taught by the current faculty. Eliminating publication requirements at such schools need not, however, remove the contributions that their faculty make to the advancement of knowledge through their research. Those faculty at such schools who are interested in research can still pursue it and publish. Moreover, such schools could support their more research-orientated faculty by providing different tracks to tenure and promotion depending on faculty interest: One with a lower teaching load but enhanced publication expectations, and one with no publication expectations but a higher teaching load. Faculty would be able to move between these tracks depending on the variance in their interests over the course of their careers. Finally, the concern that the work of teaching faculty could not be effectively assessed could be allayed by the use of different evaluative tools, as well as by the recognition that this is also a problem for the assessment of research faculty. 

## Conflict of interest

The author declares he has no conflict of interest.

## References

[1] Bryant J, Comisky PW, Crane JS, Zillmann D (1980). Relationship between college teachers' use of humor in the classroom and students' evaluations of their teachers. J Educ Psychol.

[2] Carroll J (2022). Woozles: who is to blame and what can be done? Reflections on Taylor’s prescriptive project. Reason Papers.

[3] Chávez, K, Mitchell KMW (2020). Exploring bias in student evaluations: gender, race, and ethnicity. PS: Polit Sci Polit.

[4] Collie RJ, Granziera H, Martin AJ (2019). Teachers' motivational approach: Links with students’ basic psychological need frustration, maladaptive engagement, and academic outcomes. Teach Teacher Educ.

[5] Costas R, Bordons M (2007). The h-index: Advantages, limitations and its relation with other bibliometric indicators at the micro level. J Informetrics.

[6] Department of Biology, The College of New Jersey (2021). Disciplinary standards for reappointment, tenure, and promotion. Department of Biology, The College of New Jersey.

[7] Leek JT, Taub MA, Pineda FJ (2011). Cooperation between referees and authors increases peer review accuracy. PLoS One.

[8] Mengel, F, Sauermann J, Zölitz U (2019). Gender bias in teaching evaluations. J Eur Econ Assoc.

[9] Pöschl U, Koop T (2008). Interactive open access publishing and collaborative peer review for improved scientific communication and quality assurance. Inf Serv Use.

[10] Tang BL (2023). The underappreciated wrong of Aigiarism – bypass plagiarism that risks propagation of erroneous and bias content. EXCLI J.

[11] Taylor JS (2022). Markets with limits: how the commodification of academia derails debate.

[12] Teixeira da Silva JA, Dobránszki J (2018). Rejoinder to “Multiple versions of the h-index: cautionary use for formal academic purposes”. Scientometrics.

[13] White AE (2023). Limits to markets with limits: an examination of James Stacey Taylor, Markets with limits: how the commodification of academia derails debate. Int J App Phil.

